# Hematopoietic Stem Cell Transplantation: Recent Advances

**DOI:** 10.3390/jcm14103379

**Published:** 2025-05-13

**Authors:** Giuseppe Milone, Salvatore Leotta, Alessandra Cupri

**Affiliations:** Program for Hematopoietic Stem Cells Transplantation, Azienda Ospedaliera Universitaria Policlinico di Catania, 95100 Catania, Italy; leotta3@yahoo.it (S.L.); alessandracupri@hotmail.it (A.C.)

Hematopoietic progenitor cell transplantation was the first form of cell therapy to be developed [1,2] and is still a cornerstone in treating hematologic malignancies [3,4]. One limitation of allogeneic transplantation is its toxicity, and many complications may follow the transplant; these complications affect various organs and, in some cases, may be severe enough to determine treatment-related mortality [5].

Initially, toxicity was thought to be caused essentially by the high doses of radio-chemotherapy administered [6], and in addition, it was shown that the activation of the coagulation pathway [7] and endothelial dysfunction [8] were contributing factors. Later, it became clear that alloreactivity is a crucial determinant of endothelial dysfunction after allogeneic HSCT [8,9].

This Special Issue of *JCM*, dedicated to advancements in HSCT, includes fifteen papers, one of which is an extensive review of the mechanisms of endothelial damage after allogeneic transplantation [10]. This review also highlights treatments with new agents, such as drugs which are active on the Ang 1/Ang 2 axis, moderators of thrombin activation, thrombomodulin, and the pleiotropic agent defibrotide.

Another aspect that, together with toxicity, reduces the success rate of the transplant technique is the recurrence of underlying haematological malignancies. The optimisation of the eradication conditioning phase could reduce the risk of post-transplant recurrence, as determined in a review by Jethava YS [11]. This can be attempted by incorporating into conditioning drug-conjugated monoclonal antibodies or antibody–radionuclide conjugates targeting CD45 or other antigens present in neoplastic or leukaemic cells [12,13,14]. In this *JCM* Special Issue, a study by Stoffel T et al. investigated the association of Polatuzumab vedotin, a drug-conjugated monoclonal antibody, with the high-dose and eradicating chemotherapy regimen BeAM which is used for conditioning to an autologous transplant in patients affected by relapsed or refractory NHL [15]. Polatuzumab vedotin is an anti-CD79b antibody that is conjugated with monomethyl auristatin E, a microtubule inhibitor. Although preliminary and obtained from only 12 patients affected by NHL, the results were encouraging. With a median follow-up of one and a half years, 11/12 patients were in CR. Further studies incorporating other monoclonal antibodies active in B-cell-type neoplasia are awaited.

In this *JCM* Special Issue, the results obtained from multiple myeloma patients receiving transplants in the outpatient setting are described. The study by K Larsen et al., carried out at the MM Center in Little Rock [16], described 500 of such procedures. Sixty-one percent of the patients were treated under an outpatient regime, and in these patients, the hospitalisation rate was 31.6%; the remaining portion of patients (68.4%) did not require hospitalisation. The predictive factors for admission were female sex, age, and type of conditioning. The safety of the technique was confirmed with a low TRM: 0.5% in the first 30 days in the overall series of the first autologous HSCT, whereas the TRM at 100 days was 2.9%.

One of the major advances in this field over the past decade has been the use of high-dose cyclophosphamide at an early stage after allogeneic transplantation (CTX-post), which has allowed the use of donors with limited HLA compatibility (haploidentical family donors) [17,18]. CTX-post has also broadened the number of candidates for HSCT, especially for patients whose ethnicity is under-represented in the donor registry [19]. CTX-post may be used in graft-versus-host prophylaxis from donors other than those who are haploidentical [20]. However, the advantage of CTX-post compared to other types of GVHD prophylaxis in the UD setting is not firmly established. An area that has attracted interest from many clinical investigators is the use of CTX-post in combination with anti-thymocyte globulin (ATG) [21,22,23,24]. In this Special Issue, a group from S. Raffaele Hospital reports on their experience combining, in haploidentical transplantations, a post-transplant dose of ATG with the standard dose of post-transplant cyclophosphamide [25].

Determining the most convenient donor is a topic that, over time, has increased in importance. Since CTX-post allows for the safe clinical use of a haploidentical family donor, each patient has an increased number of possible donors compared to the past, and thus, in everyday practice, the choice between unrelated donors and haploidentical donors is often encountered [26,27,28]. In this *JCM* issue, advice is provided regarding the choice of the donor [29]. According to the author, the selection criteria should differ based on the degree of donor/recipient compatibility (10/10 HLA match versus 7/8 HLA match).

Graft failure is a rare but severe complication of HSCT [30] that may be observed in sensitised patients who have high anti-HLA titres and in patients with specific underlying haematological pathologies, such as thalassemia and myelofibrosis. Intensifying pre-transplant immunosuppression is an effective way to reduce graft failure [31]. In this *JCM* issue, Mulas and colleagues [32] reviewed the conditioning of thalassemia patients, which now includes an intensive immunosuppressive phase.

Diseases based on an autoimmune mechanism are increasingly treated via autologous hematopoietic transplants. High doses of chemotherapy and autologous support have been adopted for more than 20 years in multiple sclerosis treatment, as determined in the review by H Zephir [33]. Autologous HSCT is effective in relapsing–remitting subtypes of multiple sclerosis, as highlighted in this *JCM* Special Issue [34].

In the future, the introduction of new drugs and new cellular therapies will reduce the need for autologous transplantation in the treatment of lymphomas and multiple myeloma. However, these new approaches will be limited by their high cost compared to the wide applicability of high doses of chemotherapy supported by transplantation. Furthermore, new drugs, such as bispecific monoclonal antibodies, CAR-T cells, check-point inhibitors, and, in general, all-targeted pharmacological therapies, would benefit from the existence of synergy with the graft-versus-tumour effect after allogeneic hematopoietic transplantation. Conceivably, these agents will also have synergy with autologous transplantation. Therefore, an area of clinical research that will be pursued in the future is the integration of these therapies [35,36].

Hopefully, in the coming years, other innovations will reach the stage of full clinical application. The selection and expansion of specific cellular lymphoid populations will allow for the goal of inhibiting acute GVHD and maintaining GVT to be achieved [37]. New ways of assessing alloreactivity will allow for a more precise means of using pharmacologic immunosuppressant drugs, while a better understanding of microbiota will be exploited with the aim of modulating alloreactivity [38,39].
